# Recovery of ambulation in small, nonbrachycephalic dogs after conservative management of acute thoracolumbar disk extrusion

**DOI:** 10.1111/jvim.17149

**Published:** 2024-07-25

**Authors:** Sam Khan, Nick D. Jeffery, Paul Freeman

**Affiliations:** ^1^ Department of Veterinary Medicine University of Cambridge Cambridge United Kingdom; ^2^ Department of Small Animal Clinical Studies, College of Veterinary Medicine and Biomedical Sciences Texas A&M University College Station Texas USA

**Keywords:** compression, contusion, disk herniation, spinal cord injury

## Abstract

**Background:**

Currently, low‐level evidence suggests loss of ambulation associated with acute thoracolumbar disk extrusion is best treated by decompressive spinal surgery. Conservative management can be successful, but the proportion of dogs that recover and the fate of herniated material are uncertain.

**Objectives:**

Determine the proportion of nonambulatory dogs with conservatively treated acute thoracolumbar disk extrusion that recover ambulation and measure the change in spinal cord compression during the first 12 weeks after presentation.

**Animals:**

Seventy‐two client‐owned nonambulatory dogs with acute thoracolumbar intervertebral disk extrusion.

**Methods:**

This is a prospective cohort study. Enrolled dogs underwent magnetic resonance imaging at presentation and owners were provided with conservative management recommendations. Imaging was repeated after 12 weeks. Recovery of ambulation was defined as 10 consecutive steps without falling. Spinal cord compression was determined from the cross‐sectional area of the vertebral canal and extradural compressive material at the lesion epicenter. The association between recovery and change in compression over the 12‐week observational period was examined.

**Results:**

Forty‐nine of fifty‐one (96%; 95% confidence interval [CI], 87%‐99%) of deep pain‐positive and 10/21 (48%; 95% CI, 28%‐68%) of deep pain‐negative dogs recovered ambulation within the 12‐week period. The median time to ambulation was 11 and 25 days for deep pain‐positive and ‐negative dogs, respectively. Reduction in spinal cord compression varied among individuals from minimal to complete and apparently was unrelated to the recovery of ambulation.

**Conclusions and Clinical Importance:**

A high proportion of conservatively treated dogs recovered ambulation after conservative management of acute thoracolumbar disk herniation. Recovery was not dependent on the resolution of compression.

**Abbreviations**
CI: confidence interval
CSF: cerebrospinal fluid
DP: deep pain
DPN: deep pain negative
DPP: deep pain positive
HASTE: half Fourier single‐shot turbo spin echo
IQR: interquartile range
MRI: magnetic resonance imaging
NSAIDs: nonsteroidal anti‐inflammatory drugs
PMM: progressive myelomalacia
TL IVDE: thoracolumbar intervertebral disk extrusion

## 1 INTRODUCTION

Thoracolumbar myelopathy caused by acute intervertebral disk extrusion (TL IVDE) is a common presentation in both primary care and referral practice.^1^, ^2^ Clinical signs are the result of herniation of degenerate nucleus pulposus material through the annulus fibrosus,^3^ causing mixed contusive and compressive spinal cord injuries. Although veterinarians have been aware of this type of myelopathy as long ago as the 1890s,^4^ uncertainty exists regarding the appropriate treatment for affected dogs.^5^ With the possible exception of durotomy,^6^, ^7^, ^8^, ^9^, ^10^ no current treatment directly targets the contusive injury, and thus, at present, treatment options can be categorized as either conservative or surgical.

Conservative management generally consists of analgesia, movement restriction using a crate or pen, and bladder management, with recovery dependent upon the resolution of spinal cord inflammation and mechanisms of neuroplasticity that restore function through changes in both damaged and intact pathways. However, naturally occurring resorption of extradural compressive material (extruded nucleus and hemorrhage) and consequent decompression also may play a role. This phenomenon has been reported commonly in humans^11^, ^12^, ^13^, ^14^, ^15^ and sporadically in dogs,^16^, ^17^, ^18^ but it is unclear how commonly it occurs in dogs and its relationship with recovery has not been investigated in this species. Surgical management of TL IVDE usually consists of spinal cord decompression by hemilaminectomy or mini‐hemilaminectomy, although other techniques have been described.^19^, ^20^, ^21^, ^22^ This type of management partly relies on the same recovery mechanisms as conservative management but also aims to relieve spinal cord compression and its effects on blood supply and impulse conduction.^23^, ^24^, ^25^

When presented with a dog suffering from suspected or confirmed TL IVDE, clinicians must decide whether to undertake surgical decompression. Surgical and conservative management options have not been directly compared in formal trials, but there is a consensus that for dogs that are unable to walk, surgical decompression is superior to conservative management and potentially even necessary for recovery.^26^ It also often is stated that surgery leads to more rapid and complete recovery and decreases the risk of recurrence, but these statements have not been systematically investigated.^26^ However, it is known that some dogs can recover without surgical treatment. A recent systematic review estimated that 86% of deep pain‐positive (DPP) dogs recover ambulation after conservative management.^27^ Therefore, a strong consensus in favor of surgery is potentially problematic if financial restrictions or geography limit its availability and could lead to dogs being euthanized when they might have recovered under conservative care. The aim of our prospective study was to describe the natural history of TL IVDE in conservatively managed nonambulatory dogs, and to report the proportions of both DPP and deep pain‐negative (DPN) dogs that recovered as well as time to recovery, and its association with spontaneous decompression.

## 2 MATERIALS AND METHODS

Cases were prospectively recruited from primary care and referral hospitals to the Queen's Veterinary School Hospital (QVSH) from October 2020 to the end of December 2022. Ethical approval for this study was granted by the University of Cambridge ethical committee (CR317E). No sample size calculation was completed before undertaking the study because of a lack of adequate available information from which to derive a suitable calculation and the descriptive study design. The inclusion criteria were as follows:

- Weight < 15 kg
- Nonambulatory in the pelvic limbs
- Suspected to have a TL IVDE
- Owners unable to afford surgery but able to pay the study entry fee

Nonambulatory was defined as being unable to walk a minimum of 10 steps unaided. Dogs were excluded if they had a diagnosis other than TL IVDE on magnetic resonance imaging (MRI). After the death of 1 French bulldog from aspiration pneumonia, brachycephalic dogs (French bulldogs and English bulldogs and pugs) were excluded because of increased risk of regurgitation, and ethical approval was appropriately updated.

On presentation, clinical history was used to determine the time from first clinical signs to loss of ambulation, duration of loss of ambulation before presentation and, when relevant, time from onset of clinical signs to loss of deep pain (DP) perception. Where time of loss of DP perception was not known, it was recorded as the same time as the loss of ambulation. This information was used to classify cases as peracute (<1 hour), acute (1‐24 hours), and gradual (>24 hours) onset as described previously.^28^ Subsequently, affected dogs underwent clinical and neurological examination. Information recorded included age, breed, sex, neuter status, weight, body condition score, concurrent illnesses, neuroanatomical localization, and neurologic grade, including DP perception. The severity of functional loss was graded according to the modified Frankel scale.^29^, ^30^

- Grade 3: nonambulatory paraparesis
- Grade 4: paraplegia with DPP perception
- Grade 5: paraplegia with DPN perception

Deep pain perception was evaluated by successively applying increasing pressure to the digits and metatarsal region of the pelvic limbs and tail using large artery forceps. Deep pain perception was considered intact if there was a behavioral response when pressure was applied to ≥1 of these areas. All dogs in which DP perception was thought to be absent also were assessed by a board‐certified neurologist at the time of presentation using the same method. Dogs with clinical and neurologic signs consistent with progressive ascending‐descending myelomalacia (PMM), such as flaccid pelvic limbs, pelvic limb and anal areflexia, absent cutaneous trunci reflex or a cutaneous trunci cutoff in the cranial thoracic spine, reported progression to tetraparesis or tetraplegia, inability to sit upright, and Horner's syndrome^31^, ^32^, ^33^, ^34^, ^35^ were included in outcome data but euthanized without further investigation. If possible, diagnosis was confirmed at necropsy. To ensure convincing evidence of PMM before euthanasia, dogs were hospitalized for up to 48 hours or until consistent large change in the caudal border of the cutaneous trunci reflex was noted. As a result, all affected dogs had progressed to some degree of tetraparesis before euthanasia. Necropsy confirmation of PMM secondary to a TL IVDE was not required for inclusion in the study.

All patients were hospitalized for a maximum of 2 days before being discharged and managed conservatively at home for 12 weeks. During this period, they received analgesia, cage rest, physiotherapy and, if required, bladder management. Owner support and monitoring were provided remotely via telephone, video recordings, and email. Recovery of ambulation (10 steps unaided) was self‐reported by owners during this period (as requested at the initial consultation) and supported by video recordings.

While hospitalized, all dogs received multimodal analgesia consisting of nonsteroidal anti‐inflammatory drugs (NSAIDs), acetaminophen, and gabapentin. Methadone was administered IV as rescue analgesia if required. Dogs already prescribed glucocorticoids had them discontinued. After hospitalization, patients were discharged with NSAID, acetaminophen, and gabapentin, which were gradually withdrawn over 2 to 4 weeks, depending on the response to medication.

In all cases, strict cage rest was prescribed for an initial 4 weeks, allowing on‐leash walks only for urination and defecation purposes 4 to 5 times daily. No specific instruction was provided on crate size other than its being only large enough for the dog to turn and lie down naturally. If ambulation was recovered within 4 weeks, short leash walks were initiated for a maximum of 10 minutes 4 times daily. Further less strict (large pen or room) rest beyond 4 weeks was prescribed if ambulation had not recovered. Exercise then was gradually increased throughout the 12‐week period in accordance with clinical progression.

No professional physiotherapy was provided. Instead, instruction (in person and via video tutorial) in basic massage, passive range of motion, and assisted standing exercises was provided, and these were undertaken 3 to 4 times daily. Assisted standing exercises consisted of stand‐to‐sits, sit‐to‐stands, rocking, and foot sliding exercises (see Supporting Information S1).

The bladder in each dog was managed by manual expression every 6 hours during hospitalization. If temperament did not allow for manual expression, an indwelling catheter was placed and removed at discharge. After discharge, owners were instructed to manage the bladder by manual expression 3 to 4 times daily until consistent voluntary urination had been witnessed. Time to control of urination was recorded as reported by owners, as were complications such as urinary tract infection, although routine urinalysis was not undertaken and urodynamic studies were not possible to confirm continence. Instead, urine was tested if clinical signs suggestive of infection (pyrexia, lethargy, polydipsia, and hematuria) were present.

Twelve weeks after the initial presentation, the dogs were again presented to the QVSH and each underwent full clinical and neurologic examination. Successful outcome was defined as the ability to walk 10 steps unaided, and time to ambulation was recorded as reported by the owners.

**FIGURE 1:**
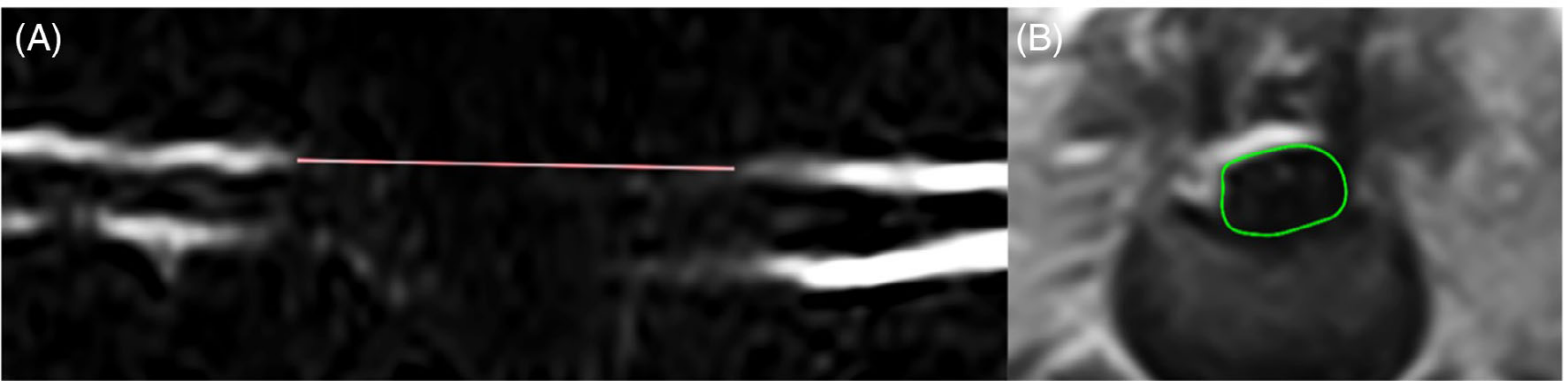
Sagittal HASTE MR image showing CSF attenuation measurement (A), and T2 transverse MR image showing cross‐sectional measurement of extradural compressive material (B). CSF, cerebrospinal fluid; HASTE, half Fourier single‐shot turbo spin echo; MR, magnetic resonance

With the exception of those presenting with signs consistent with PMM, dogs were sedated for MRI using a low field 0.27 T MRI unit (MR‐Grande, Esaote) to confirm the diagnosis of TL IVDE, using the following sequences: T2‐weighted sagittal, half Fourier single‐shot turbo spin echo sagittal, T2‐weighted transverse, and T1‐weighted transverse. Imaging was repeated 12 weeks later using the same parameters. All images were evaluated using a standard digital imaging and communication in medicine viewer (Osirix 64‐bit, Pixmeo SARL) by 1 author (SK). Using the straight‐line function, the length of attenuation of the dorsal cerebrospinal fluid (CSF) column extending cranially and caudally from the site of TL IVDE, and the length of the second lumbar vertebra (L2) were measured as previously described.^36^ The ratio of CSF column attenuation to the length of L2 was calculated. The cross‐sectional area of extradural compressive material and the vertebral canal were measured at the point of maximal compression using the closed polygon function to calculate vertebral canal compromise as previously described^37^, ^38^ (Figure 1).

Data were coded in Microsoft Excel and exported to RStudio (2020) for statistical analysis. Normality was tested using the Shapiro‐Wilk test and normal Q‐Q plots. The equality of variance was tested using Bartlett's test or Levene's test where applicable. Other assumptions were checked using residuals versus fitted, scale location and residuals versus leverage plots where appropriate. Proportion of recovery was reported as intention‐to‐treat (all dogs, including those with conclusive signs of myelomalacia, presenting for an initial consultation) and, where relevant, as per protocol (ie, minus those euthanized because of suspected PMM before MRI and therefore without confirmation of diagnosis; Figure 4).

## 3 RESULTS

**FIGURE 2:**
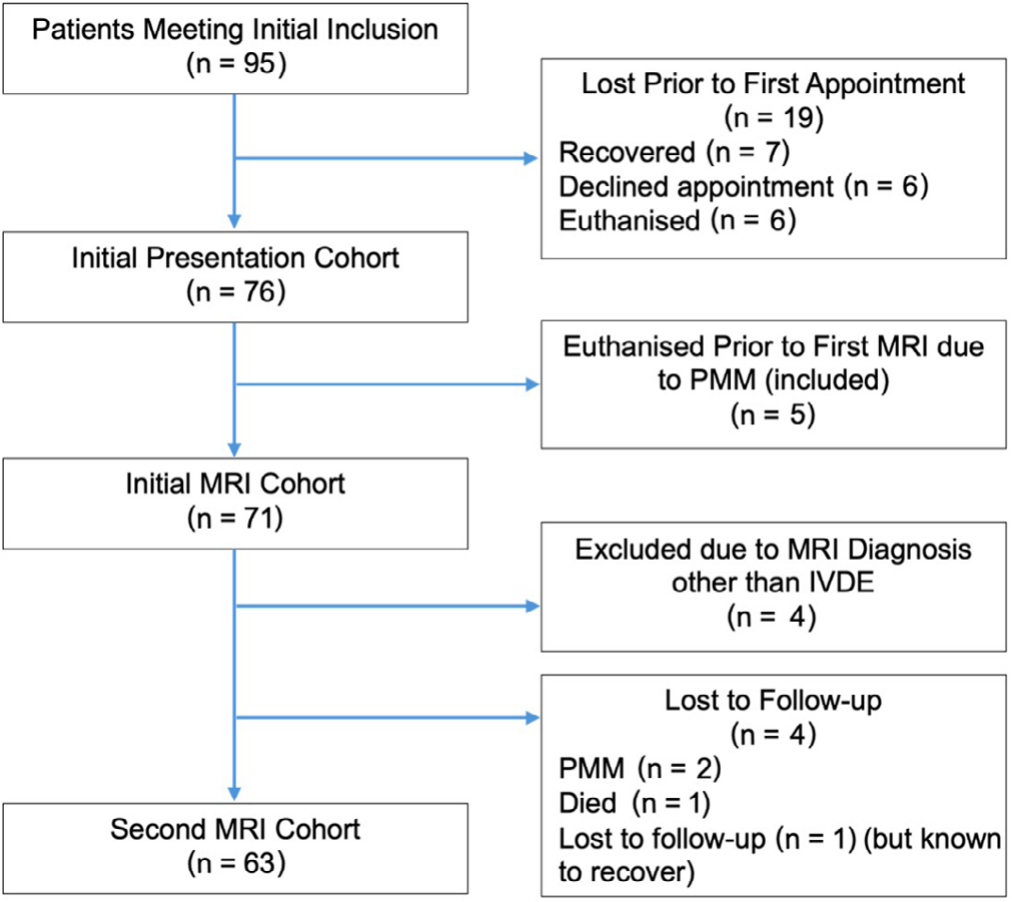
Flow chart of patient inclusion and exclusion

Ninety‐five cases met the initial inclusion criteria. Of these, 19 were lost before their first appointment because of either recovering ambulation (n = 7), declining an appointment because of financial cost (£525 participation fee), transport restrictions (n = 6), or being euthanized (n = 6). No further data are available for these cases. Therefore, the initial cohort consisted of 76 cases, with 5 euthanized before MRI because of suspected PMM, and a further 4 excluded because of having an MRI diagnosis other than TL IVDE. Thus, 67 cases were confirmed by MRI and, of these, 4 did not return for repeat MRI leaving a 2nd MRI cohort of 63 (Figure 2).

Fifty‐one of 72 were DPP on presentation, and 21 of 72 were DPN on presentation, including 5 with suspected PMM.

Demographic data are presented in Table 1.

**TABLE 1:** Demographic data for the initial analysis cohort

Most common breeds	Miniature dachshund (n = 57) Standard dachshund (n = 5) Crossbreed (n = 5)
Sex	Male, n = 30 (17 neutered) Female, n = 42 (27 neutered)
Age	Median = 60 months (IQR, 52‐80)
Weight	Median = 6.6 kg (IQR, 5.5‐7.8)
Rate of onset	Peracute (<1 hour), n = 3 Acute (1‐24 hours), n = 42 Gradual (>24 hours), n = 26
Time from loss of ambulation to presentation	Median = 5 days (IQR, 3‐7)

Abbreviation: IQR, interquartile range.

## 4 DEEP PAIN POSITIVE COHORT (N = 51)

Demographic data for this cohort are presented in Table 2.

**TABLE 2:** Demographic data for the deep pain positive cohort

Most common breeds	Miniature dachshund (n = 39) Standard dachshund (n = 5) Crossbreed (n = 3)
Sex	Male, n = 24 (14 neutered) Female, n = 27 (20 neutered)
Age	Median = 64 months (IQR, 58‐84)
Weight	Median = 6.7 kg (IQR, 5.5‐8.3)
Rate of onset	Peracute (<1 hour), n = 2 Acute (1‐24 hours), n = 26 Gradual (>24 hours), n = 22
Time from loss of ambulation to presentation	Median = 5 days (IQR, 3‐8)
Most common sites of disk extrusion	T13‐L1 (n = 13) T12‐13 (n = 12) L2‐3 (n = 7)

Abbreviation: IQR, interquartile range.

**FIGURE 3:**
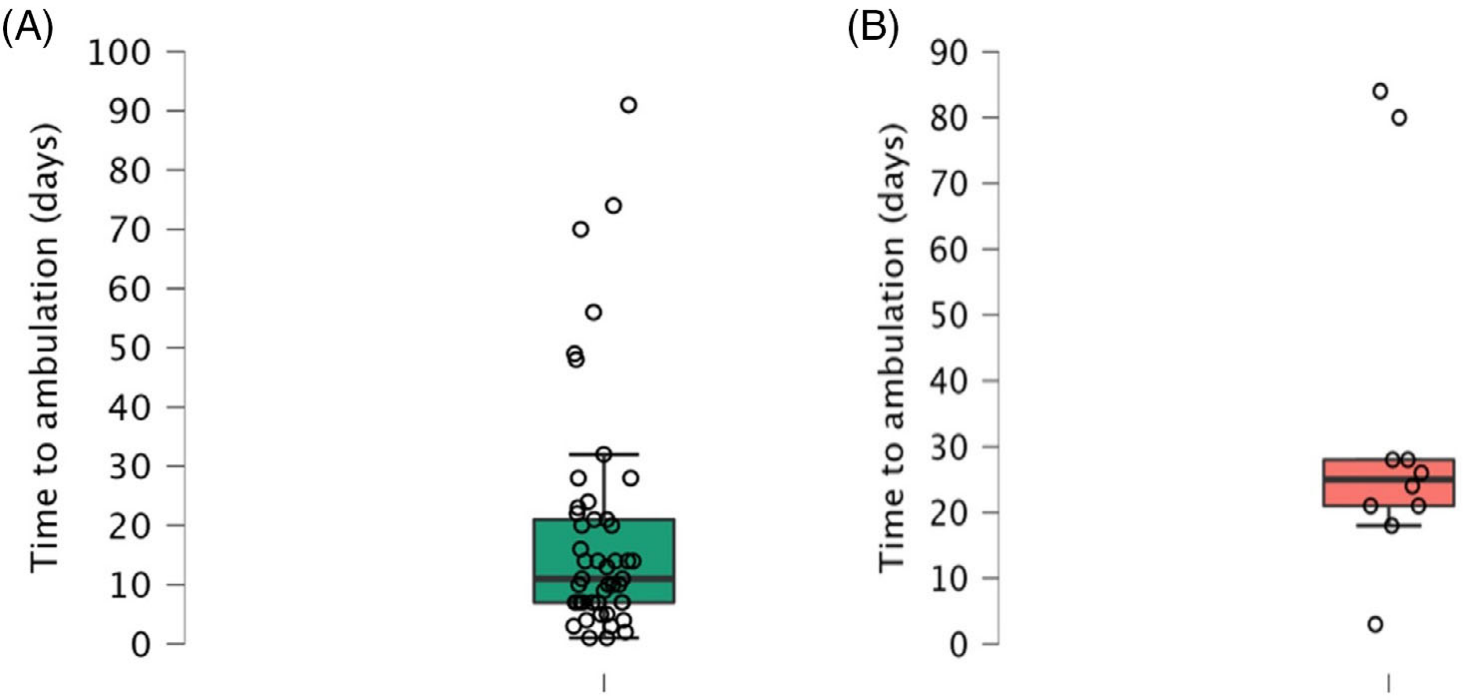
A combined scatter and box and whisker plot showing time to ambulation in DPP (A) and DPN (B) dogs as individual data points (circles), median, quartiles (box), and upper and lower extremities (bars). DPN, deep pain negative; DPP, deep pain positive

Within the 12‐week study period, 49/51 (96%; 95% confidence interval [CI], 87%‐99%) DPP dogs recovered ambulation. The median time to recovery of ambulation was 11 (interquartile range [IQR], 7‐21; range, 1‐91) days (Figure 3). Of the 2 dogs that failed to recover, 1 died of suspected aspiration pneumonia after initial MRI and 1 showed no neurological improvement but remained DPP. At follow‐up, none of the dogs had persistent back pain and all those that recovered ambulation also recovered voluntary urinary function. In 2 dogs, clinical signs recurred; 1 with recurrence of back pain alone, and PMM in the other. Repeat MRI confirmed that these outcomes were the result of a second intervertebral disk extrusion at a different site in both cases.

## 5 DEEP PAIN NEGATIVE OUTCOME (N = 21)

Demographic data are presented in Table 3.

**TABLE 3:** Demographic data for the deep pain negative cohort

Most common breeds	Miniature dachshund (n = 18) Crossbreed (n = 2) Lhasa apso (n = 1)
Sex	Male, n = 6 (3 neutered) Female, n = 15 (7 neutered)
Age (months)	Median = 58 (IQR, 48‐66)
Weight (kg)	Median = 6.1 (IQR, 5.5‐7.0)
Rate of onset	Peracute (<1 hour), n = 1 Acute (1‐24 hours), n = 16 Gradual (>24 hours), n = 4
Time from loss of ambulation to presentation (days)	Median = 4 (IQR, 3‐6)
Most common sites of disk extrusion	T11‐12 (n = 6) T13‐L1 (n = 4) T12‐13 (n = 3)

Abbreviation: IQR, interquartile range.

**FIGURE 4:**
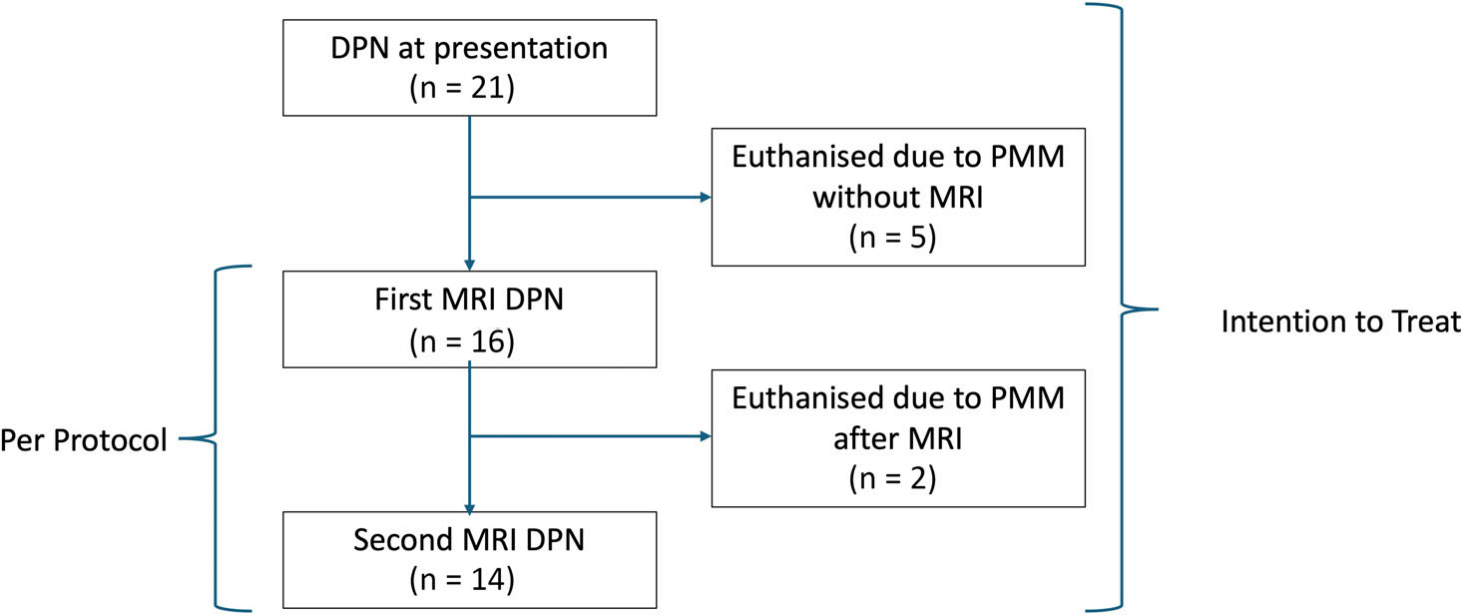
A flow chart tracing DPN dogs through the study period. DPN, deep pain negative

Of the 21 dogs that were DPN on presentation (ie, intention to treat; Figure 4) 10/21 (48%; 95% CI, 28‐68) recovered ambulation with a median time to ambulation of 25 (IQR, 21‐28; range, 3‐84) days (Figure 3), and 7 had developed PMM on presentation or later (33%; 95% CI, 17‐55). Excluding the 5 dogs (Figure 4) that were euthanized before the initial MRI because of apparent PMM (ie, per protocol), the recovery and PMM rates were 63% (95% CI, 39%‐82%) and 13% (95% CI, 4%‐36%), respectively. The 4 dogs that did not recover ambulation and survived follow‐up remained DPN. At follow‐up, no dogs had persistent back pain and all dogs that recovered ambulation also recovered voluntary urinary function. Nonambulatory paraparesis recurred in 1 dog but repeat MRI confirmed that it was the result of a second intervertebral disk extrusion at a different site.

## 6 CHANGE IN COMPRESSION

**FIGURE 5:**
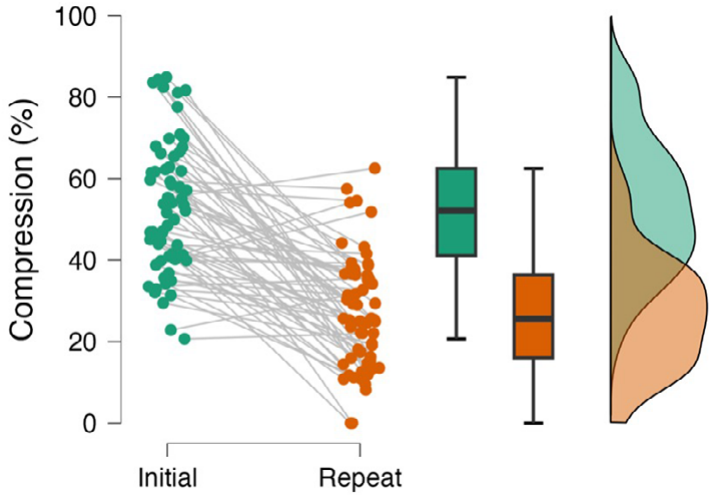
A scatter plot, box plot, and raincloud plot showing compression visible on initial and repeat imaging

**FIGURE 6:**
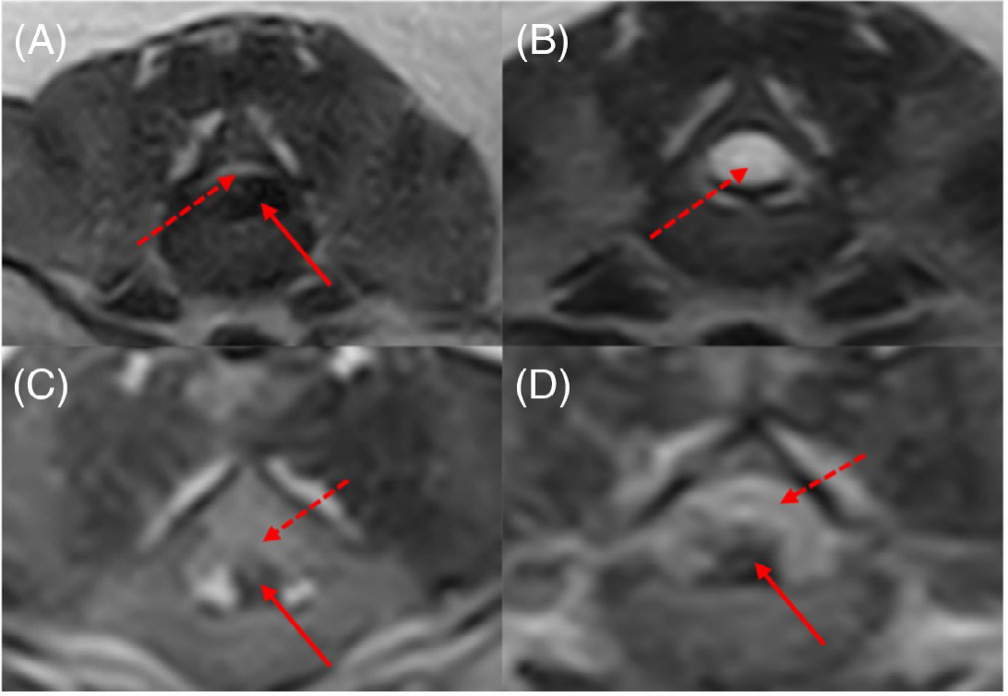
Transverse T2‐weighted images showing near‐complete regression of extradural compressive material (solid arrows) between 0 (A) and 12 weeks (B) and little to no change (C and D). The spinal cord is annotated using dotted arrows. Note the hyperintense spinal cord (B) suspected to be gliosis

Mean compression on initial and repeat MRI were 53% ± 16% and 28% ± 14%, respectively. Median absolute reduction in compression (initial %‐repeat %) was 22% (IQR, 3‐37; Figure 5). Some dogs showed complete resolution (n = 2), whereas others showed <5% change (n = 9; Figure 6).

**FIGURE 7:**
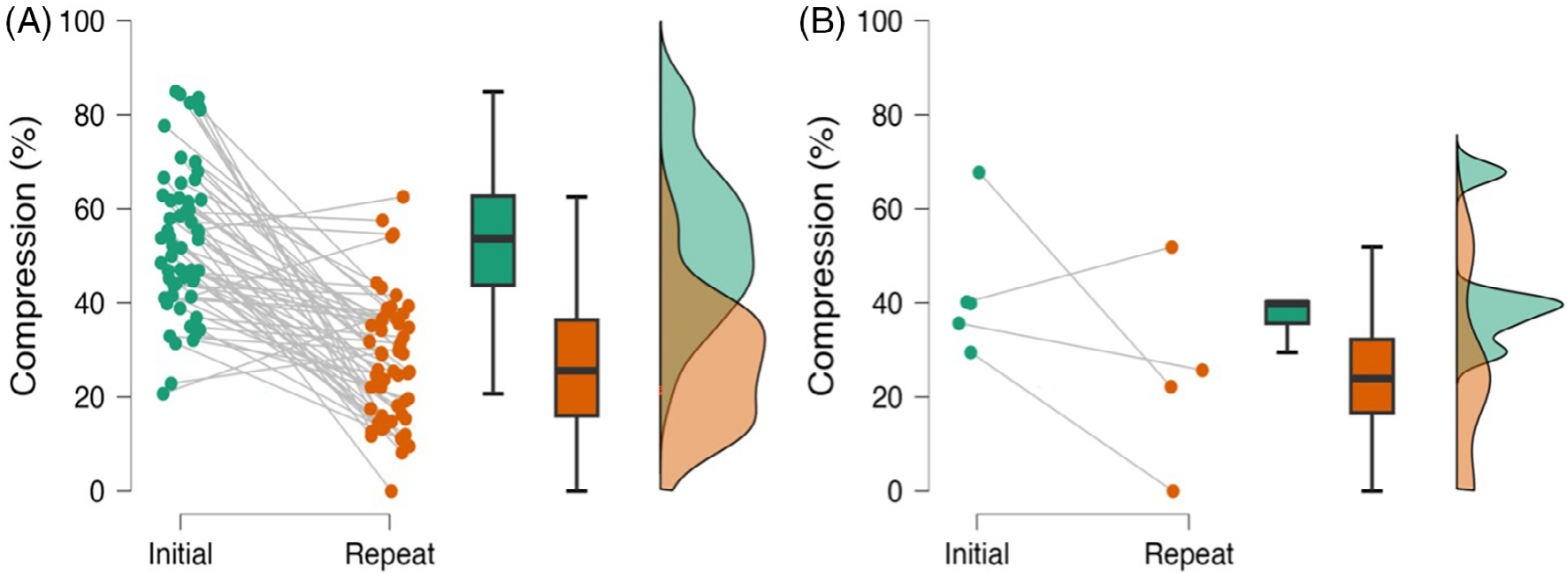
Scatter plots, box plots, and raincloud plots showing compression visible on initial and repeat imaging for those that recovered (A) and those that did not (B)

**FIGURE 8:**
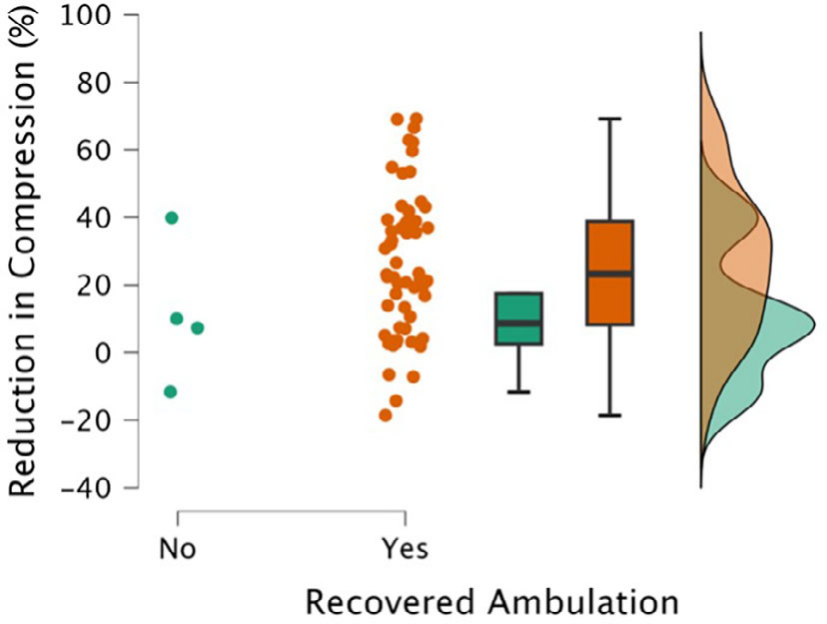
A combined scatter, box, and raincloud plot of the reduction in compression (initial %‐repeat %) by recovery of ambulation

When subdivided by outcome, both groups experienced some reduction in compression, with some showing near‐complete elimination of extradural material and some remaining unchanged (Figures 7 and 8).

## 7 DISCUSSION

Our results augment the existing evidence that a large proportion of dogs that are unable to walk after acute TL IVDE can recover ambulation within 12 weeks of the original injury without surgical intervention. Almost all dogs retaining DP perception recovered, as well as approximately 50% of DPN dogs.

Previous reports and systematic reviews had reported a favorable prognosis for DPP dogs managed conservatively (89% and 65%),^27^, ^39^ which is corroborated by our findings, although the recovery rate reported in our study is slightly higher than previously described.^27^, ^39^ The outcome for DPN dogs was substantially better than previous reports of conservative management,^27^, ^39^, ^40^ although a 60% recovery rate has been recorded previously.^41^ The reason for the disparity between our study and other conservative management studies is unclear, but previous reports were retrospective, and therefore, the reasons why dogs were assigned to conservative management or details regarding the excluded populations are not always clear, inevitably implying selection bias, and making the results difficult to generalize to a wider population. Alternatively, our population may have had less severe cellular injury, which could have been identified using serum biomarkers. However, given the wide range of compression and CSF attenuation, this possibility is unlikely to entirely explain the difference reported here. Another possible reason is that, in some studies, dogs were re‐directed to surgical management after 4 weeks if they had not recovered from ambulation.^42^ In our dataset, several dogs recovered >28 days after presentation, especially in the DPN cohort, and in such circumstances would have been classified as not recovering in previous reports when they might have recovered if given more time.

The proportions of dogs that recover in this cohort are similar to those reported after decompressive surgery (96% vs approximately 90% DPP and 48% vs approximately 50% DPN),^26^, ^27^, ^39^, ^40^ with considerable overlap of the CIs reported here and in surgical studies, adding to the evidence that surgical management is not the sole route to recovery for many nonambulatory dogs.^5^ Importantly, the time to presentation in this investigation is similar to that of several previous reports of surgical treatment.^30^, ^34^, ^36^, ^43^

The median time to recovery for DPP dogs was not dissimilar to a previous report (18 days)^44^ but did differ substantially from others that reported recovery times of 63 days for grade 3 and 84 days for grade 4.^39^ This was not repeated for the DPN cohort in which recovery time (25 days) was slightly longer than the 18 days described in a previous meta‐analysis.^39^ However, both are similar to previous reports after decompressive surgery^34^, ^45^ although the definitions of ambulation were different. Because of the different methods used to define ambulation and the subjective nature of many of the techniques, it is difficult to draw meaningful conclusions regarding the disparity in recovery time, although all previous studies reported a wide range of recovery times, which in itself is an important finding, because many dogs in the past have been determined to not have recovered after a relatively short time period.

Here, we record a higher frequency of PMM (33%) than in many other reports,^6^, ^31^, ^32^, ^35^, ^46^, ^47^ except those focused only on French bulldogs.^48^ There are many possible reasons for this discrepancy, but perhaps the most likely is that we included dogs that already had evidence of PMM at presentation and these may have been omitted in many similar reports.

Our study is the first to systematically report the naturally occurring change in spinal cord compression after TL IVDE with time. Our data show an overall substantial reduction in compression over a 12‐week period, although with considerable variation among individuals. Interestingly, the resolution of compression and persistent compression was evident in both dogs that recovered and those that did not. Therefore, the role of spontaneous decompression in the recovery of dogs managed conservatively remains unclear and may play a larger role in some individuals than others.

Our study had several limitations. First, the compressive material seen on MRI was not confirmed to be extruded nucleus pulposus. In theory, it is possible that a substantial proportion of the visible compression on MRI was related to hemorrhage. However, given the signalment of the dogs, the onset and progression of the clinical signs, and the MRI characteristics, this is unlikely. Instead, it is much more likely that the compressive material was a mixture of extruded nucleus pulposus and hemorrhage and that both hemorrhage and nucleus were removed over the 12 weeks. Analysis of the severity of cord compression is hindered by a lack of complete duplication of the location of transverse images between the 2 timepoints, and some difference in measured compression may be caused by that factor alone. It is also possible that the findings may not be applicable to brachycephalic dogs or those >15 kg (because they were not included in our study), but the majority of dogs with TL IVDE will be <15 kg and most studies have not found an association with weight and outcome.^47^, ^49^, ^50^

In conclusion, our results augment evidence that a large proportion of small dogs with TL IVDE will recover without surgical intervention, including many of those who are DPN and those with severe spinal cord compression. It also confirms that disk extrusions in dogs can regress, but that regression and spontaneous decompression are not necessarily required for functional recovery. Combining these 2 findings indicates that if surgical decompression is not possible because of financial or geographic restrictions, conservative management is a valid alternative. Although experience tells us that some dogs affected by TL IVDE recover only after surgical decompression, our results suggest the need for further investigation into clinical and MRI characteristics to help determine exactly which dogs fall into this group.

## CONFLICT OF INTEREST DECLARATION

Authors declare no conflict of interest.

## OFF‐LABEL ANTIMICROBIAL DECLARATION

Authors declare no off‐label use of antimicrobials.

## INSTITUTIONAL ANIMAL CARE AND USE COMMITTEE (IACUC) OR OTHER APPROVAL DECLARATION

Approved by the Department of Veterinary Medicine, University of Cambridge (CR317E).

## HUMAN ETHICS APPROVAL DECLARATION

Authors declare human ethics approval was not needed for this study.

## Supporting information


**Data S1.** Supporting Information.

## References

[1] Bergknut N , Egenvall A , Hagman R , et al. Incidence of intervertebral disk degeneration‐related diseases and associated mortality rates in dogs. J Am Vet Med Assoc. 2012;240(11):1300‐1309. 22607596 10.2460/javma.240.11.1300

[2] Fluehmann G , Doherr MG , Jaggy A . Canine neurological diseases in a referral hospital population between 1989 and 2000 in Switzerland. J Small Anim Pract. 2006;47(10):582‐587. 17004950 10.1111/j.1748-5827.2006.00106.x

[3] Hansen HJ . A pathologic‐anatomical study on disc degeneration in dog, with special reference to the so‐called enchondrosis intervertebralis. Acta Orthop Scand Suppl. 1952;11:1‐117. 14923291 10.3109/ort.1952.23.suppl-11.01

[4] Olsson SE . Observations concerning disc fenestration in dogs. Acta Orthop. 1951;20(4):349‐356. 10.3109/17453675108991182 14894205

[5] Freeman P , Jeffery N . Is decompression in acute thoracolumbar intervertebral disc herniation overvalued? Front Vet Sci. 2022;9:1‐5. 10.3389/fvets.2022.1049366 PMC9682007 36439357

[6] Takahashi F , Honnami A , Toki M , et al. Effect of durotomy in dogs with thoracolumbar disc herniation and without deep pain perception in the hind limbs. Vet Surg. 2020;49(5):860‐869. 32166788 10.1111/vsu.13409

[7] Hirano R , Asahina R , Hirano T , Hyakkoku A , Miura R , Kunihiro T . Outcomes of extensive hemilaminectomy with durotomy on dogs with presumptive progressive myelomalacia: a retrospective study on 34 cases. BMC Vet Res. 2020;1–9. 33287802 10.1186/s12917-020-02690-z PMC7720392

[8] Jeffery ND , Mankin JM , Ito D , et al. Extended durotomy to treat severe spinal cord injury after acute thoracolumbar disc herniation in dogs. Vet Surg. 2020;49(5):884‐893. 32277768 10.1111/vsu.13423

[9] Saadoun S , Jeffery ND . Acute traumatic spinal cord injury in humans, dogs, and other mammals: the under‐appreciated role of the dura. Front Neurol. 2021;12(February):1‐7. 10.3389/fneur.2021.629445 PMC7887286 33613434

[10] Jeffery ND , Rossmeisl JH , Harcourt‐brown TR , et al. Randomized controlled trial of durotomy as an adjunct to routine decompressive surgery for dogs with severe acute spinal cord injury. 2024;5:128‐138. 10.1089/neur.2023.0129 PMC10898236 38414780

[11] Autio RA , Karppinen J , Niinimäki J , et al. Determinants of spontaneous resorption of intervertebral disc herniations. Spine (Phila Pa 1976). 2006;31(11):1247‐1252. 16688039 10.1097/01.brs.0000217681.83524.4a

[12] Komori H , Okawa A , Haro H , Muneta T , Yamamoto H , Shinomiya K . Contrast‐enhanced magentic resonance imaging in conservative management of lumbar disc herniation. Spine (Phila Pa 1976). 1998;23(1):67‐73. 9460155 10.1097/00007632-199801010-00015

[13] Chiu CC , Chuang TY , Chang KH , Wu CH , Lin PW , Hsu WY . The probability of spontaneous regression of lumbar herniated disc: a systematic review. Clin Rehabil. 2015;29(2):184‐195. 25009200 10.1177/0269215514540919

[14] Guinto FC , Hashim H , Stumer M . CT demonstration of disk regression after conservative therapy. Am J Neuroradiol. 1984;5(5):632‐633. 6435432 PMC8335144

[15] Saal J , Saal J , Herzog R . The natural history of umbar intervertebral disc extrusions treated nonoperatively. Spine (Phila Pa 1976). 1990;15(7):683‐686. 2218716 10.1097/00007632-199007000-00013

[16] Steffen F , Kircher PR , Dennler M . Spontaneous regression of lumbar Hansen type 1 disk extrusion detected with magnetic resonance imaging in a dog. J Am Vet Med Assoc. 2014;244(6):715‐718. 24568114 10.2460/javma.244.6.715

[17] Khan S , Freeman P , Jeffery ND . Spontaneous resolution of severe disc‐associated spinal cord compression in a dog. J Small Anim Pract. 2022;63(10):797. 35729741 10.1111/jsap.13526

[18] Argent V , Fraser A , Alves L , Freeman P . Spontaneous regression of a cervical intervertebral disc extrusion in French bulldogs documented on MRI after medical management. Vet Rec Case Rep. 2019;7(2):1‐4.

[19] Flegel T , Neurology DA , Boettcher IC , et al. Partial lateral corpectomy of the thoracolumbar spine in 51 dogs: assessment of slot morphometry and spinal cord decompression. Vet Surg. 2011;40:14‐21. 21077918 10.1111/j.1532-950X.2010.00747.x

[20] Funkquist B . Decompressive laminectomy in thoraco‐lumbar disc protrusion with paraplegia in the dog. J Small Anim Pract. 1970;11:445‐451. 5530006 10.1111/j.1748-5827.1970.tb05595.x

[21] Gage ED , Hoerlein BF . Hemilaminectomy and dorsal laminectomy for relieving compressions of the spinal cord in the dog. J Am Vet Med Assoc. 1968;152(4):351‐359. 5761375

[22] Salger F , Vet M , Ziegler L , et al. Neurologic outcome after thoracolumbar partial lateral corpectomy for intervertebral disc disease in 72 dogs. Vet Surg. 2014;43:1‐8. 24484371 10.1111/j.1532-950X.2014.12157.x

[23] Al‐Mefty O , Harkey HL , Marawi I , Peeler DF , Haines DE , Alexander LF . Experimental chronic compressive cervical myelopathy: effects of decompression. J Neurosurg. 1995;83(2):336‐341. 7616281 10.3171/jns.1995.83.2.0336

[24] Dimar JR 2nd , Glassman SD , Raque GH , Zhang YP , Shields CB . The influence of spinal canal narrowing and timing of decompression on neurologic recovery after spinal cord contusion in a rat model. Spine (Phila Pa 1976). 1999;24(16):1623‐1633. 10472095 10.1097/00007632-199908150-00002

[25] Jeffery ND , Blakemore WF . Spinal cord injury in small animals 1. Mechanisms of spontaneous recovery. Vet Rec. 1999;144:407‐413. 10331228 10.1136/vr.144.15.407

[26] Olby NJ , Moore SA , Brisson B , et al. ACVIM consensus statement on diagnosis and management of acute canine thoracolumbar intervertebral disc extrusion. J Vet Intern Med. 2022;36(5):1570‐1596. 35880267 10.1111/jvim.16480 PMC9511077

[27] Freeman P , Jeffery ND . Re‐opening the window on fenestration as a treatment for acute thoracolumbar intervertebral disc herniation in dogs. J Small Anim Pract. 2017;58(4):199‐204. 28276121 10.1111/jsap.12653

[28] Scott HW , Mckee WM . Laminectomy for 34 dogs with thoracolumbar intervertebral disc disease and loss of deep pain perception. J Small Anim Pract. 1999;40(9):417‐422. 10516947 10.1111/j.1748-5827.1999.tb03114.x

[29] Frankel HL , Hancock DO , Hyslop G , et al. The value of postural reduction in the initial management of closed injuries of the spine with paraplegia and tetraplegia part I. Paraplegia. 1969;7(3):179‐192. 5360915 10.1038/sc.1969.30

[30] Scott HW . Hemilaminectomy for the treatment of thoracolumbar disc disease in the dog: a follow‐up study of 40 cases. J Small Anim Pract. 1997;38(11):488‐494. 9403807 10.1111/j.1748-5827.1997.tb03303.x

[31] Balducci F , Canal S , Contiero B , Bernardini M . Prevalence and risk factors for presumptive ascending/descending myelomalacia in dogs after thoracolumbar intervertebral disk herniation. J Vet Intern Med. 2017;31(2):498‐504. 28144987 10.1111/jvim.14656 PMC5354033

[32] Gilmour LJ , Jeffery ND , Miles K , Riedesel E . Single‐shot turbo spin echo pulse sequence findings in dogs with and without progressive myelomalacia. Vet Radiol Ultrasound. 2017;58(2):197‐205. 27977066 10.1111/vru.12463

[33] Griffiths IR . The extensive myelopathy of intervertebral disc protrusions in dogs (‘the ascending syndrome’). J Small Anim Pract. 1972;13:425‐437. 5081200 10.1111/j.1748-5827.1972.tb06870.x

[34] Jeffery ND , Barker AK , Hu HZ , et al. Factors associated with recovery from paraplegia in dogs with loss of pain perception in the pelvic limbs following intervertebral disk herniation. J Am Vet Med Assoc. 2016;248(4):386‐394. 26829270 10.2460/javma.248.4.386

[35] Okada M , Kitagawa M , Ito D , Itou T , Kanayama K , Sakai T . Magnetic resonance imaging features and clinical signs associated with presumptive and confirmed progressive myelomalacia in dogs: 12 cases (1997‐2008). J Am Vet Med Assoc. 2010;237(10):1160‐1165. 21073387 10.2460/javma.237.10.1160

[36] Ito D , Matsunaga S , Jeffery ND , et al. Prognostic value of magnetic resonance imaging in dogs with paraplegia caused by thoracolumbar intervertebral disk extrusion: 77 cases (2000‐2003). J Am Vet Med Assoc. 2005;227(9):1454‐1460. 16279391 10.2460/javma.2005.227.1454

[37] Huska JL , Gaitero L , Brisson BA , Nykamp S , Thomason J , Sears WC . Presence of residual material following mini‐hemilaminectomy in dogs with thoracolumbar intervertebral disc extrusion. Can Vet J. 2014;55(10):975‐980. 25320387 PMC4187364

[38] Gupta A , Upadhyaya S , Yeung CM , et al. Disk area is a more reliable measurement than anteroposterior length in the assessment of lumbar disk herniations: a validation study. Clin Spine Surg. 2020;33(8):E381‐E385. 32149746 10.1097/BSD.0000000000000958

[39] Langerhuus L , Miles J . Proportion recovery and times to ambulation for non‐ambulatory dogs with thoracolumbar disc extrusions treated with hemilaminectomy or conservative treatment: a systematic review and meta‐analysis of case‐series studies. Vet J. 2017;220:7‐16. doi:10.1016/j.tvjl.2016.12.008 28190499

[40] Olby NJ , da Costa RC , Levine JM , Stein VM . Prognostic factors in canine acute intervertebral disc disease. Front Vet Sci. 2020;7(November):1‐14. 33324703 10.3389/fvets.2020.596059 PMC7725764

[41] Joaquim JGF , Luna SPL , Brondani JT , Torelli SR , Rahal SC , De Freitas FP . Comparison of decompressive surgery, electroacupuncture, and decompressive surgery followed by electroacupuncture for the treatment of dogs with intervertebral disk disease with long‐standing severe neurologic deficits. J Am Vet Med Assoc. 2010;236(11):1225‐1229. 20513202 10.2460/javma.236.11.1225

[42] Levine JM , Levine GJ , Johnson SI , Kerwin SC , Hettlich BF , Fosgate GT . Evaluation of the success of medical management for presumptive thoracolumbar intervertebral disk herniation in dogs. Vet Surg. 2007;36(5):482‐491. 17614930 10.1111/j.1532-950X.2007.00295.x

[43] Aikawa T , Fujita H , Kanazono S , Shibata M , Yoshigae Y . Long‐term neurologic outcome of hemilaminectomy and disk fenestration for treatment of dogs with thoracolumbar intervertebral disk herniation: 831 cases (2000‐2007). J Am Vet Med Assoc. 2012;241(12):1617‐1626. 23216037 10.2460/javma.241.12.1617

[44] Han H , Yoon H , Kim J , et al. Clinical effect of additional electroacupuncture on thoracolumbar intervertebral disc herniation in 80 paraplegic dogs. Am J Chin Med. 2010;38(6):1015‐1025. 21061457 10.1142/S0192415X10008433

[45] Rosen S , Lynn J , Schocke C , et al. A 50‐step walking test for analysis of recovery after decompressive surgery for thoracolumbar disc herniation in dogs. J Vet Intern Med. 2022;36(5):1733‐1741. 36161381 10.1111/jvim.16516 PMC9511074

[46] Fenn J , Ru H , Jeffery ND , et al. Association between anesthesia duration and outcome in dogs with surgically treated acute severe spinal cord injury caused by thoracolumbar intervertebral disk herniation. J Vet Intern Med. 2020;34(4):1507‐1513. 32418346 10.1111/jvim.15796 PMC7379036

[47] Olby NJ , Levine J , Harris T , Muñana KR , Skeen T , Sharp N . Long‐term functional outcome of dogs with severe injuries of the thoracolumbar spinal cord: 87 cases (1996–2001). J Am Vet Med Assoc. 2003;222(6):762‐769. 12675299 10.2460/javma.2003.222.762

[48] Aikawa T , Shibata M , Asano M , Hara Y , Tagawa M , Orima H . A comparison of thoracolumbar intervertebral disc extrusion in French bulldogs and dachshunds and association with congenital vertebral anomalies. Vet Surg. 2014;43:301‐307. 24433331 10.1111/j.1532-950X.2014.12102.x

[49] Davis GJ , Brown DC . Prognostic indicators for time to ambulation after surgical decompression in nonambulatory dogs with acute thoracolumbar disk extrusions: 112 cases. Vet Surg. 2002;31:513‐518. 12415519 10.1053/jvet.2002.36015

[50] Ruddle TL , Allen DA , Schertel ER , et al. Outcome and prognostic factors in non‐ambulatory Hansen type I intervertebral disc extrusions: 308 cases. Vet Comp Orthop Traumatol. 2006;19(1):29‐34. 16594541

