# Supplementary material for: Recovery of ambulation in small, nonbrachycephalic dogs after conservative management of acute thoracolumbar disk extrusion
Source: J Vet Intern Med. 2024 Jul 25;38(5):2603–11. doi: 10.1111/jvim.17149 (PMC11423491; doi:10.1111/jvim.17149)
Supplement: Supplementary file 1 — Data S1. Supporting Information. [file JVIM-38-2603-s001.pdf]

NAME

PHYSIOTHERAPY

In the hospital, **Name** has been receiving physiotherapy. This should be continued at home to maintain her muscle mass and strength and encourage healthy blood flow to her tissues. Ideally it is best if you can manage three to four sessions each day, but we do realise that this is not always possible. If you are short of time, then "little and often" is better than one long session.

While she has mainly been receiving physiotherapy on her hind limbs, you are more than welcome to repeat the exercises on her fore limbs if you wish to do so. The physiotherapy is taking us approximately 10-15 minutes each side (followed by approximately 5 minutes of the standing exercises), but again, don't panic if you need to make these sessions shorter.

Her physiotherapy instructions are as follows:

Massage ~ Assist **Name** to lie on her side, and support (with your hand or a towel) the limb you will be massaging, so that the joints are not put under any strain.

1. Begin with "***effleurage***" (a stroking motion) at the start of a session to try and relax and warm the bodily tissues. Generally, massage in the direction of the coat, unless you have been instructed to do otherwise due to any swelling that your dog may have. Beginning with light pressure, use both hands in a sweeping/stroking motion, covering all target muscles, in order to create a continuous motion, rather than stop-start. Gradually increase the pressure as the tissue starts to warm. Lightly skim over any bony prominences. Spend **5 minutes** performing effleurage on each intended area.
2. Then perform a **kneading massage technique**, working on the areas that have already received effleurage. Make small circles with your fingers or hold muscle between fingers and thumb and make a kneading motion (almost like making breadcrumbs with your fingers). Spend a further **5 minutes** performing this kneading on each target area.

This warm-up is important to avoid excessive strain on the muscles during the remainder of the physiotherapy session.

PROM (Passive Range of Motion) ~ Once the muscles have been warmed up, slowly and gently flex and extend each limb **6-8 times**. This exercise should take a further **2 to 3 minutes**. There are two types of PROM that you should perform:

- **Concertina movement:** Supporting at least two joints (e.g. knee and hock in the hind limb), flex and extend the limb towards and away from the body.

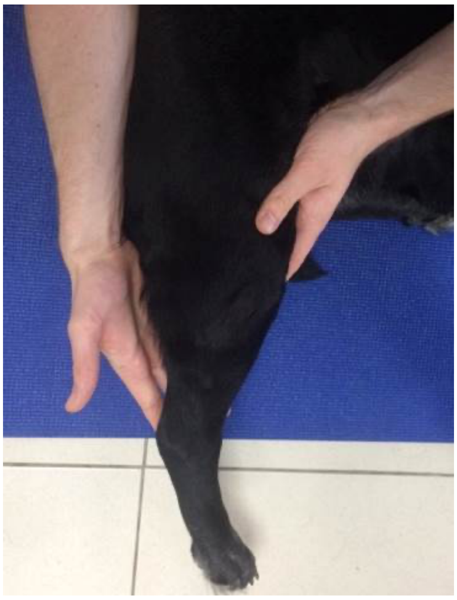

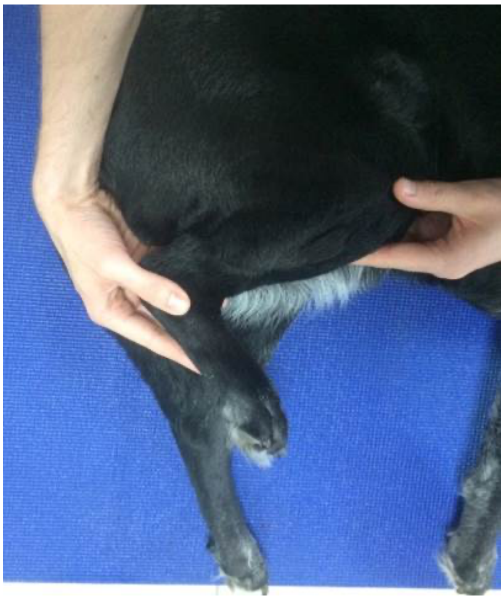

- **Bicycle movement:** Supporting at least two joints. Flex the limb up, extend forward so that the limb is pointing towards the fore limbs and then guide it back so that it is now pointing backwards whilst keeping the limb straight (i.e. all movement coming from the hip). Return to the neutral/starting position before flexing up and repeating.

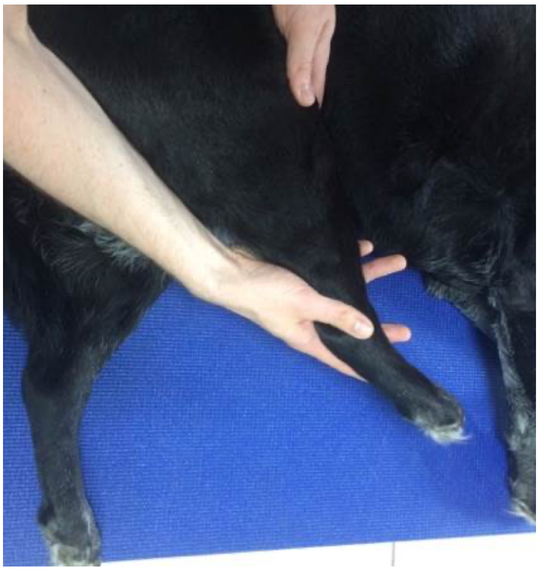

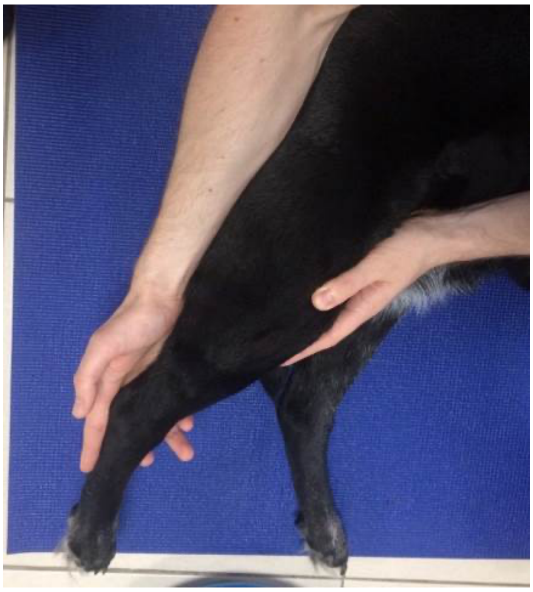

It is very important that the joints are not pushed beyond their normal range of movement – if you feel there is any stiffness, just ease off a little, hold the position for approximately five seconds, and then relax the position. The next time you reach that point in the joint movement, you may find that the range of movement is better than before. It is very important that you never force a movement when you feel resistance as this may tear a muscle or ligament. Make sure to keep the limb in line with the body and parallel to the floor and do not lift it past horizontal. This is especially important for patients suffering from hip dysplasia and/or arthritis. These exercises will help to maintain flexibility and reduce stiffness.
Effleurage ~ end each session with **2 minutes** of effleurage as a "warm down" to maintain good blood flow to the area.

Assisted standing exercises ~ Assist ***Name*** into a natural standing position on a non-slip surface (carpet, grass, etc.). Her limbs should be placed with feet squarely on the ground, in order for the limbs to take her weight. ***Name*** should be encouraged to take as much weight as possible, so that you are only providing support necessary to maintain the standing position.

You may need to include periods of rest interspersed between sets of assisted standing if you feel ***Name*** is tiring quickly. However, you should aim to slowly increase the length of time that they spend standing.

This can also be adapted to a sitting position, if you have been instructed to do so.

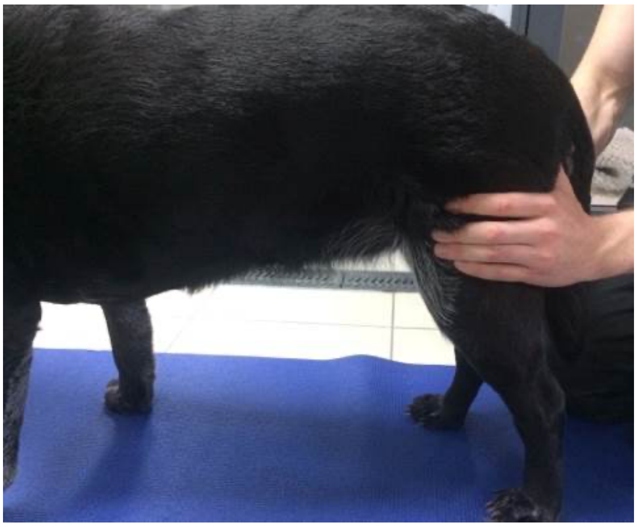

If ***Name*** is taking her own weight well, **½ sit to stands** can also be done. With a treat on the end of her nose, encourage her to sit onto a cushion or half way down your thigh. As ***Name*** gets stronger, she can sit further down your thigh, until she is doing full sit to stands straight to the floor. Perform **3 to 5 repetitions**, keeping hands on the side of her hind limbs to prevent them from splaying out to the side.

Rocking and foot sliding exercises ~ This can be done on either stable surface, such as the floor or a table, or on an unstable surface, such as a hot water bottle filled with cold water. When performing this, make sure to support ***Name*** around the hip and thigh. Make very small movements in three directions: side to side, forward and backwards and up and down.
Spend roughly 10 seconds in each direction, repeated 3 to 4 times, giving a total of around 2 minutes. If she is taking the weight well, you can continue the rocking motion further by gently lifting the opposite limb off the ground to encourage her to take more weight on the limb she is left standing on. Hold this position for several seconds before repeating the exercise in the other direction. **Repeat this five times each side**.

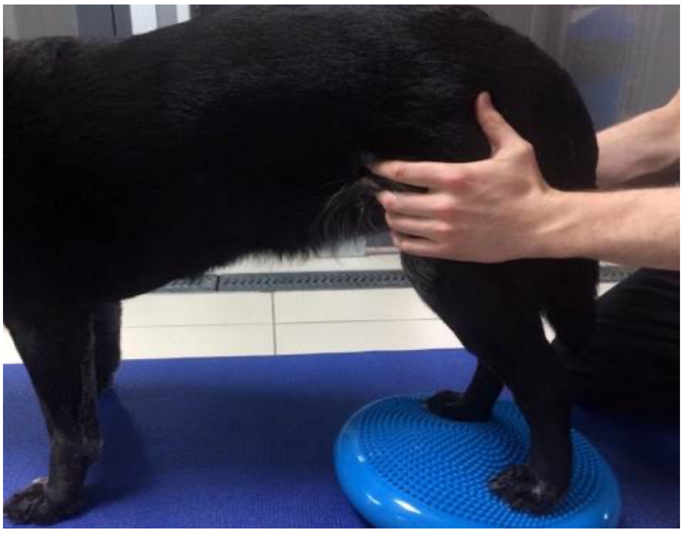

After the rocking exercises, use the opposite hand to the limb you are focusing on, to support the knee i.e. if treating right hind limb, use left hand to support the knee. Use the other hand on ***Name's*** foot to guide the limb through the motion (similar motion to bicycle PROM movement). Gentle pressure should be put on the top of her foot whilst sliding the paw over the rough side of the hot water bottle. ***Name*** may require a gentle toe pinch or pressure between the patient's pads in order for her to flex the limb to start the exercise again. **Perform 6 to 8 repetitions on each leg**.

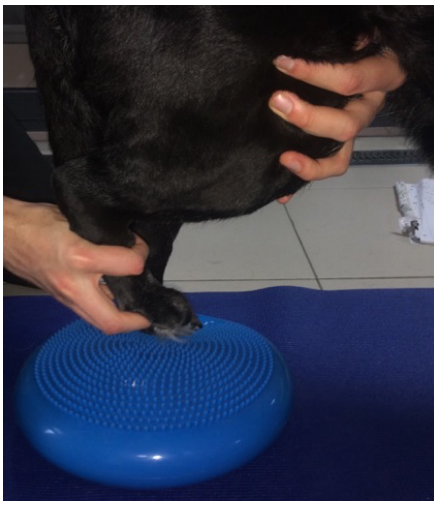

It

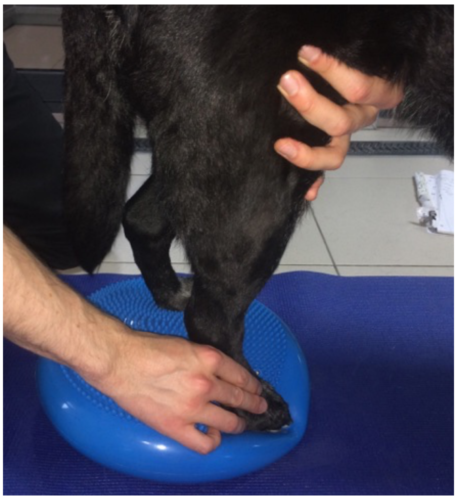

is

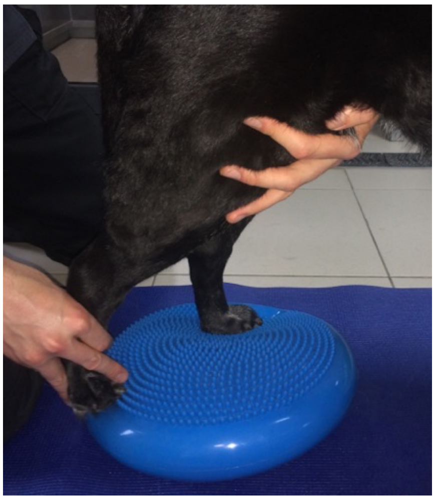

important that **Name** is encouraged to place her limbs in normal positions and she may need some reminding of this. If her toes are knuckling over when she walks, place them back in a normal position for her - likewise when she is lying down.

The main aims of these exercises are to:

1. keep her limbs flexible so that they are able to function normally.
2. re-educate her brain on how to walk again by providing stimulus from touch, pressure and movement.
3. slowly build her muscle strength and endurance.

If **Name** is dragging her toes, it is very important that you check her toes at least twice daily for any grazes, swelling or discharges. Please avoid walking **Name** on abrasive surfaces where possible. If you notice any wounds, or signs of infection or inflammation, please contact a veterinary surgeon as further treatment may be necessary.

As ***Name's*** spinal cord has been damaged, the signals from her brain to her hind limbs are slower than they were before the injury. Therefore, when taking her out for toilet breaks, it is important to maintain a slow walk to allow her hind limbs to catch up with her fore limbs and to encourage her to place them properly. If you are keeping ***Name*** in a room with a slippery floor (laminate, vinyl, etc.), please put down some non-slip rubber backed matting or carpet to provide a surface for her to grip on to when you take her out. This also reduces the chance that she will slip and potentially injure herself further.
